# Supplementary material for: Production and Transfer of Energy and Information in Hamiltonian Systems
Source: PLoS One. 2014 Feb 28;9(2):e89585. doi: 10.1371/journal.pone.0089585 (PMC3938839; doi:10.1371/journal.pone.0089585)
Supplement: Information S1 — (PDF) [file pone.0089585.s001.pdf]

Supporting Information

Information S1

Here, we prove the other main result of this paper which is the inequality:

$$I_c^{KP} < I_c < H_{\rm KS} \tag{1}$$

and thus explain the result of Fig. 3 of the manuscript. In Ref. [1], the authors discuss about the existence of the spectrum of the Lyapunov exponents in the thermodynamic limit and investigate numerically this existence in the FPU-β model given by Eq. (3) in the manuscript. They show that the shape of the Lyapunov spectrum for energy densities $\epsilon = E/N$ well above the equipartition threshold allows someone to express $H_{\rm KS}$ in terms of the largest Lyapunov exponent λ_1 only:

$$H_{\rm KS} = \int_0^{\lambda_1} \lambda C N d\lambda = \frac{N}{2}\lambda_1, \tag{2}$$

where $C = 1/\lambda_1$.

By applying the above ideas in our case for $E \in (E_{\rm u}, E_{\rm r})$ and using Eq. (2) we have:

$$\begin{aligned} H_{\rm KS} &= \int_0^{\lambda_1} \lambda C N d\lambda \Rightarrow & (3)\\ H_{\rm KS} &= \int_0^{\lambda_{N/2}} \lambda C N d\lambda + \int_{\lambda_{N/2}}^{\lambda_1} \lambda C N d\lambda \Rightarrow \\ H_{\rm KS} &= \frac{N}{2\lambda_1}\left(\lambda^2\right)_0^{\lambda_{N/2}} + \tilde{H} \Rightarrow \\ \tilde{H} &= H_{\rm KS} - \frac{N}{2\lambda_1}\left(\lambda_{N/2}\right)^2, & (4) \end{aligned}$$

where we have used $C = 1/\lambda_1$ and $\bar{H} = \int_{\lambda_{N/2}}^{\lambda_1} \lambda C N d\lambda$. Term $\lambda_{N/2}$ is the $(N/2)$th positive Lyapunov exponent of Hamiltonian (3) of the manuscript when sorting them in descending order (i.e. $\lambda_1 > \lambda_2 > \ldots > \lambda_{N/2} > \ldots > \lambda_N = 0$). It comes from the fact that in Eq. (3) we integrate over all positive Lyapunov exponents and that we want to relate $H_{\rm KS}$ with $\tilde{H}$ of Eq. (18) of the manuscript which is defined as the sum over the first $N/2$ positive Lyapunov exponents when they are sorted in descending order.

By substituting Eq. (4) in Eq. (18) of the manuscript we have:

$$\begin{aligned} I_c &= 2\tilde{H} - H_{\mathrm{KS}} \Rightarrow \\ I_c &= H_{\mathrm{KS}} - \frac{N}{\lambda_1}\left(\lambda_{N/2}\right)^2, \end{aligned} \tag{5}$$

and so we obtain:

$$I_c < H_{\mathrm{KS}} \tag{6}$$

which is the right hand side inequality of Eq. (1).

By combining Eqs. (2), (5) and setting $I_c^{KP} = \lambda_1$, we obtain:

$$\begin{aligned} I_c &= \frac{N}{2}\lambda_1 - \frac{N}{\lambda_1}\left(\lambda_{N/2}\right)^2 \Rightarrow \\ I_c &= \frac{N}{2} I_c^{KP} - \frac{N}{I_c^{KP}}\left(\lambda_{N/2}\right)^2. \end{aligned} \tag{7}$$

The last equation links the upper bound of information transfer in the phase space of the Hamiltonian with the upper bound of the information that can be transferred in the KP space. Moreover, an important consequence of Eq. (5) is that $I_c = H_{\mathrm{KS}}$ when $\lambda_{N/2} = 0$ implying that this can happen when there are at least $N/2$ integrals of motion and leading to the conclusion that it should be $\lambda_{N/2} = \lambda_{(N/2)+1} = \ldots = \lambda_N = 0$. However, this is not happening in our case since all Lyapunov exponents are positive but the last one $\lambda_N = 0$ as the Hamiltonian is an integral of the motion.

Next, we prove the left hand side inequality of Eq. (1):

$$I_c^{KP} < I_c. \tag{8}$$

To do so, let us suppose that:

$$I_c - I_c^{KP} = 0 \tag{9}$$

and check under which assumptions for I_c^{KP} Eq. (8) holds. For this, we substitute Eq. (7) for I_c into Eq. (9) and have:

$$\left(\frac{N-2}{2}\right)\left(I_c^{KP}\right)^2 - N\left(\lambda_{N/2}\right)^2 = 0. \tag{10}$$

The last equation is a second degree polynomial with respect to I_c^{KP}. Its determinant is given by:

$$\mathcal{D} = 2N(N-2)\big(\lambda_{N/2}\big)^2,$$

which is positive for $N > 2$ and thus, the two discrete real roots are:

$$\begin{aligned} I_c^{KP} &= \frac{\lambda_{N/2}\sqrt{2N(N-2)}}{N-2} > 0 \text{ and} \\ I_c^{KP} &= -\frac{\lambda_{N/2}\sqrt{2N(N-2)}}{N-2} < 0. \end{aligned} \tag{11}$$

By theory, we know that Eq. (10) is positive and thus inequality in Eq. (8) is true when $I_c^{KP} > \frac{\lambda_{N/2}\sqrt{2N(N-2)}}{N-2}$ since the term $\frac{N-2}{2}$ of I_c^{KP} is positive for $N > 2$.

The second root is not physically possible to exist since it would imply that $I_c^{KP} < 0$ for $N > 3$ contradicting to the fact that I_c^{KP} is positively defined. Thus, Eq. (9) is positive when $I_c^{KP} > \frac{\lambda_{N/2}\sqrt{2N(N-2)}}{N-2}$, which is always true, since $\lambda_{N/2} \ll 1$ and:

$$\lim_{N\to\infty} \frac{\sqrt{2N(N-2)}}{N-2} = \sqrt{2}.$$

Thus, we have proved that:

$$I_c^{KP} < I_c. \tag{12}$$

Combining Eqs. (6) and (12), we obtain:

$$I_c^{KP} < I_c < H_{\text{KS}}. \tag{13}$$

The way I_c is defined (see Eq. (18) of the manuscript) implies that $I_c < H_{\text{KS}}$ since $\tilde{H} < H_{\text{KS}}$. In panel A of Fig. 3 of the manuscript we can check that indeed inequality (13) is fulfilled.

Finally, it worths mentioning that according to Eq. (11) it is possible to have:

$$I_c^{KP} = I_c$$

that is, the upper bounds of information transfer in the bi-dimensional subspace and in the Hamiltonian

to be equal when it happens that:

$$I_c^{KP} = \frac{\lambda_{N/2}\sqrt{2N(N-2)}}{N-2} = \sqrt{2}\lambda_{N/2}.$$

The last equation provides an alternative estimation of I_c^{KP} valid when:

$$I_c^{KP} = \lambda_1 = \sqrt{2}\lambda_{N/2}.$$

References

1. Livi R, Politi A, Ruffo S (1986) Distribution of characteristic exponents in the thermodynamic limit. J Phys A: Math Gen 19: 2033-2040.
